# microRNAs and Gene–Environment Interactions in Autism: Effects of Prenatal Maternal Stress and the *SERT* Gene on Maternal microRNA Expression

**DOI:** 10.3389/fpsyt.2021.668577

**Published:** 2021-07-05

**Authors:** David Q. Beversdorf, Ayten Shah, Allison Jhin, Janelle Noel-MacDonnell, Patrick Hecht, Bradley J. Ferguson, Danielle Bruce, Michael Tilley, Zohreh Talebizadeh

**Affiliations:** ^1^Departments of Radiology, Neurology, and Psychological Sciences, William and Nancy Thompson Endowed Chair in Radiology, University of Missouri, Columbia, MO, United States; ^2^Interdisciplinary Neuroscience Program, University of Missouri, Columbia, MO, United States; ^3^Children's Mercy Hospital, Kansas City, MO, United States; ^4^Kansas City University, Kansas City, MO, United States; ^5^Children's Mercy Hospital and University of Missouri-Kansas City School of Medicine, Kansas City, MO, United States; ^6^Health Psychology, Radiology, and Thompson Center for Autism and Neurodevelopmental Disorders, University of Missouri, Columbia, MO, United States; ^7^Department of Biology, Central Methodist University, Fayette, MO, United States

**Keywords:** autism spectrum disorder, prenatal stress, miRNA, epigenetics, dopamine, gene x environment

## Abstract

**Background:** Genetics and environment both are critical in autism spectrum disorder (ASD), but their interaction (G × E) is less understood. Numerous studies have shown higher incidence of stress exposures during pregnancies with children later diagnosed with ASD. However, many stress-exposed mothers have unaffected children. The serotonin transporter (*SERT*) gene affects stress reactivity. Two independent samples have shown that the association between maternal stress exposure and ASD is greatest with maternal presence of the *SERT* short (S)-allele (deletion in the promoter region). MicroRNAs play a regulatory role in the serotonergic pathway and in prenatal stress and are therefore potential mechanistic targets in this setting.

**Design/methods:** We profiled microRNA expression in blood from mothers of children with ASD, with known stress exposure during pregnancy. Samples were divided into groups based on *SERT* genotypes (LL/LS/SS) and prenatal stress level (high/low).

**Results:** Two thousand five hundred mature microRNAs were examined. The ANOVA analysis showed differential expression (DE) of 119 microRNAs; 90 were DE in high- vs. low-stress groups (stress-dependent). Two (miR-1224-5p, miR-331-3p) were recently reported by our group to exhibit stress-dependent expression in rodent brain samples from embryos exposed to prenatal stress. Another, miR-145-5p, is associated with maternal stress. Across *SERT* genotypes, with high stress exposure, 20 significantly DE microRNAs were detected, five were stress-dependent. These microRNAs may be candidates for stress × *SERT* genotype interactions. This is remarkable as these changes were from mothers several years after stress-exposed pregnancies.

**Conclusions:** Our study provides evidence for epigenetic alterations in relation to a G × E model (prenatal maternal stress × *SERT* gene) in ASD.

## Introduction

The developmental origins of health and disease (DOHaD) hypothesis proposes that environment experiences during development *in utero* influence health after birth (1). Numerous studies demonstrate that adverse environmental exposures affect neurological development, including those which are salient to autism spectrum disorder (ASD). Genetics is a critical factor in ASD (2–6). However, importance of environmental factors is being increasingly recognized (7), with heritability estimated at 0.87 by the latest, more conservative analysis (8). While progress has been made toward the understanding of genetic factors, including development of animal models based on these genes (9–12), environmental factors are less understood.

Psychological stress during pregnancy impacts behavioral and developmental outcomes in humans (13). Early personality development in the child, schizophrenia, and emotional disturbances in offspring are all associated with maternal stress (14–17). Relationships between maternal stress and a range of adverse offspring behavioral outcomes are reported in animal models, including abnormal behavioral fear responses, as well as abnormal physiological stress reactivity in offspring, which lasts into adulthood (18, 19). Recent evidence suggests that among environmental factors, maternal stress exposure is important in ASD (20–22).

Early studies surveyed for history and timing of prenatal psychosocial stressors corresponding to major life events on the Social Readjustment Rating Scale in mothers of children with ASD, Down syndrome, and typically developing controls. A higher overall incidence of stressors among the mothers who had a child with ASD was found compared to other groups, with a peak in stressors among mothers with a child with ASD at 25–28 weeks of gestation, but not in the other groups (21). This has subsequently been supported by other studies, showing a relationship between the incidence and severity of tropical storms in the United States in Louisiana during the 5th to 6th months of gestation and the incidence of ASD births (22). Larger epidemiological studies support this relationship between prenatal stress and ASD. One Danish national registry study suggested against the presence of an association between maternal bereavement and ASD (23), but the association between maternal bereavement and ASD was present when maternal psychological conditions were included (23). Another Danish national registry study found that maternal psychological conditions were one of the strongest prenatal risk factors for ASD (24). A Swedish registry study also confirmed a relationship between third-trimester prenatal stress and ASD (25). Furthermore, results from the Nurses' Health Study, another large registry study, showed that maternal exposure to partner abuse during pregnancy is strongly associated with ASD (26). Children *in utero* in New York City during the September 11 terrorist attacks were also found to be 7–9% more likely to be in special education classes (27), although no specific data was available regarding ASD diagnoses. A recent meta-analysis has further supported the relationship between prenatal stress and ASD (28). Finally, a recent study reported that children with ASD that were exposed to prenatal stress present with a more severe condition than those with no history of prenatal stress exposure (29). Therefore, a better understanding of the relationship between prenatal stressors and gene × environment (G × E) interaction in ASD would represent a significant breakthrough, as reviewed recently (30, 31).

A significant proportion of stress-exposed mothers have unaffected children. Several factors could interact with stress exposure to increase impact on neurodevelopment. For example, prenatal exposure to air pollution, which is associated with ASD (32–35), can impact microglia–neuron interactions in a sex-specific manner (36), which may interact with prenatal stress exposure and further impact development. A potential reason why prenatal stressors might result in neurodevelopmental effects only in some cases could be a G × E interaction. One candidate is the serotonin transporter (*SERT*) gene, which is well-studied for its role in stress susceptibility. The *SERT* gene encodes for the SERT protein, which transports serotonin from the synaptic cleft back to the presynaptic neuron (37). Variations in this gene can alter aspects of its function (38–42). The most widely studied variation is an insertion or deletion of 44 base pairs within the promoter region of the *SERT* gene, *SLC6A4*, resulting in a long (L) or short (S) allele, respectively (37, 38, 40). The relationship between the S-allele (SS or LS genotype) and increased risk of depression after exposure to stress has not reliably been demonstrated (43–45). However, presence of the S-allele is also related to suicidality (46), alcoholism (47, 48), and susceptibility to anxiety (40), and greater activation of the amygdala, a brain region which is critical for fear reactions (49). The S-allele of the *SERT* gene has not been consistently linked to ASD itself (50–54), but its role as a gene mediating stress susceptibility remains of interest. It has also been recently demonstrated that maternal serotonin concentrations affect core symptoms and cognitive ability in ASD, with the lowest maternal serotonin levels associated with the greatest severity (55). Linkage studies have also associated rigid-compulsive behaviors in patients with ASD with the region of the genome containing *SERT* (56). A variation in a single nucleotide on the gene, Gly56Ala, is also linked to ASD (42). However, the *SERT* polymorphisms most reliably associated with ASD result in overactivity, rather than a loss of function. Therefore, for a potential role of the S-allele in a G × E interaction in ASD, the superimposed effect of prenatal stress on the maternal *SERT* genotype in this case may be distinct from the mechanism of the *SERT* polymorphisms directly associated with ASD.

The clinical salience of a G × E interaction was explored for *SERT* and prenatal stress exposure, demonstrating that the relationship between prenatal stress exposure and ASD appears to be mediated by maternal genetic susceptibility to stress, specifically by maternal presence of the S-allele, which has been shown in two independent patient populations (57). Stress surveys were administered to two independent samples of mothers of children with ASD. The *SERT* gene was genotyped for the L-alleles and S-alleles in the mothers. Those individuals that have the LS or SS genotypes (~64% of the population) are known from previous work (43, 45–49) to have increased stress reactivity and express lower levels of *SERT* than those individuals that have the L/L genotype. Thus, mothers were examined for the presence vs. absence of the S-allele as a genetic marker associated with stress reactivity and the presence vs. absence of prenatal stress according to stress surveys (57).

If the S-allele is a maternal risk factor for development of ASD with exposure to prenatal stress, one might expect that among mothers of children with ASD, a history of prenatal stress exposure during pregnancy would be observed more frequently in presence of the S-allele. In other words, if the association between prenatal stress and ASD was driven by the need for additional presence of maternal genetic susceptibility to stress, then this association between prenatal stress and ASD should be primarily observed in the pregnancies of mothers with this genetic susceptibility to stress. In both samples, the presence of the S-allele and the history of prenatal stress were found to significantly co-segregate in mothers of children with ASD within the critical period of pregnancy suggested in our previous work. To account for the possibility that the S-allele simply conferred increased recall of stress, or for some other reason greater exposure to during pregnancy, prenatal stress exposure history was obtained from the same mothers for the pregnancies of the proband's unaffected sibling. There was no increase in report of prenatal stress exposure regardless of genotype when these same mothers were queried about pregnancies of unaffected siblings. This suggests that the S-allele does not cause an overall increase in recall of stress or stress exposure during pregnancy. Rather, this provides support that the S-allele might serve as a genetic risk factor for increased maternal stress response in association with the development of ASD, and the effect is specific to exactly the same timeframe as reported by previous research using self-report measures and also the timeframe suggested by the Louisiana storms (21, 22, 57).

An animal model was developed to facilitate exploration of mechanism and experimental manipulations directed at developing treatment and prevention strategies. Social behavior was examined in the offspring of female *SERT*-heterozygous knockout (*SERT*-het) mice, whose SERT function is reduced 50%, comparable to that observed in humans with the S-allele and are known to have an increased susceptibility to stress (41, 58, 59), which are then exposed to chronic variable stress during gestation. In a 2 ×2 (stress × genotype) experimental design, *SERT*-wild-type (*SERT*-wt) and *SERT*-het dams were exposed to stress during gestation. A control group for each genotype had no stress exposure. This stress paradigm has previously been shown effective based on cortisol measurements but does not cause changes in feeding or body weight (60). Using the three-chamber social approach task (61), a significant maternal genotype × stress interaction was found, with unstressed offspring of wild-type mice spending significantly more time with the novel stranger than prenatally stressed offspring of *SERT*-het dams, supportive of a maternal gene/stress interaction in offspring behavior in the mouse model. These offspring of stressed heterozygous mothers did not demonstrate more general anxiety as assessed by elevated plus maze, suggesting specificity of this avoidance effect to the social domain (62).

To explore the mechanism of these effects, epigenetic markers were explored. Recent research revealed numerous gene expression changes associated with stress exposure. With maternal stress exposure in rodents, placental tissue showed increased expression in peroxisome proliferator-activated receptors α (PPARα), insulin-like growth factor-binding protein 1 (IGFBP-1), GLUT4, and HIF3α, specifically observed in placentas associated with male offspring (63), in addition to O-GlcNAc transferase (OGT) (64), of particular interest given the high percentage males with ASD. MicroRNAs (miRNAs) also play a significant regulatory role in serotonergic pathways (65, 66), immunity (67), and prenatal stress (68–70). Dysregulation of miR-103, miR-145, miR-219, miR-323, and miR-98 is associated with maternal stress (71). Inflammatory responses in the brain may be altered by miR-323 and miR-98 (71). MiR-135 regulates response to chronic stress through interaction with serotonergic activity (72). Furthermore, roles of specific miRNAs have been reported in regulating serotonergic genes (Let-7a) (66), *SERT* (miR-16 and miR-15a) (73, 74), and *SLC6A4* (miR-325) (75). Recent work has shown that parental stress effects on offspring are also mediated by miRNA changes (76, 77).

Previous work explored the miRNA gene profile, expression, and methylation profile in the brains of the offspring of this *SERT*-het/stress model in mice, revealing a striking attenuation of the gene expression and miRNA changes in response to stress in the brains of the *SERT*-het/stress offspring mice in contrast to response to prenatal stress in the brains of *SERT*-wt mice (78). Significantly increased global methylation was observed in *SERT*-het/stress offspring brains, and there were more upregulated miRNA in stressed control mice as compared to wt, but not for *SERT*-het/stress compared to *SERT*-wt. Similarly, there were fewer upregulated genes when *SERT*-het/stress was compared to *SERT*-wt than when stressed control mice were compared to wt. Therefore, with increased methylation (generally suppressing gene expression), and decreased miRNA and overall expression, it appears that the typical epigenetic response to stress in offspring brains is blunted in the presence of maternal *SERT*-het (78).

Given the findings in the G × E mouse model, we began to explore the potential for detecting epigenetic changes in the clinical G × E setting. As an initial investigation in this direction, we examined the miRNA profile in the samples from the mothers in the previously described clinical G × E study to determine if significant changes were detectable in those mothers of children with ASD exposed to stress during pregnancy and whether these were further impacted by the maternal *SERT* genotype. Additionally, we performed exome sequencing of the maternal samples in an exploratory manner to determine whether there are other maternal factors that might be of interest for potential future exploration.

## Methods

### Samples

Thirty-four maternal blood samples from one of the sites in the previous G × E study were examined in this study. We did not have any samples with LS genotype in the low-stress group. Therefore, the samples were divided into five groups based on *SERT* genotypes and prenatal stress level, as shown in Table 1. Families with a child diagnosed with ASD under the age of 10 years were contacted from the University of Missouri Thompson Center for Autism & Neurodevelopmental Disorders database. All participants with ASD were below 10 years of age (average age = 6.8 ± 1.8) to maximize the parents' ability to recall information from the prenatal period. Families were invited to provide samples for genetic analysis and complete a questionnaire regarding the prenatal period. All ASD diagnoses were confirmed *via* Autism Diagnostic Interview-Revised (ADI-R) (79) and/or Autism Diagnostic Observation Scale (ADOS) (80) scores. Experimental procedures were approved and conducted in accordance with the University of Missouri Health Sciences Institutional Review Board. Blood was drawn *via* a standard venipuncture from the median cubital vein of the arm. Genomic DNA was obtained from the subjects' whole blood (FlexiGene Kit; Qiagen, Hilden, Germany) according to the manufacturer's instructions. PCR was performed as previously described (81). Briefly, the promoter region of the serotonin transporter gene was amplified using the Qiagen PCR kit from 25 ng genomic DNA to determine the *SERT* genotype, as described in a previous study (57), with 35% of mothers revealing the SS genotype, 32% the SL genotype, and 32% the LL genotype from the sample in the present study, where samples from mothers with at least three stressors were selected for the high-stress group, and samples from mothers with 0 or 1 stressor were selected for the low-stress group. At the time of the appointment with the experimenter, mothers completed questionnaires regarding their child with ASD as well as the gestational period in which the stressors occurred for that child. Details of the stress surveys to which the mothers replied are previously described (57). Survey questions obtained information on the child's birth date, pregnancy length, and the occurrence and subjective severity of major stressful events during the pregnancy. A list of common stressors was provided to the subjects to facilitate recall of events that may have occurred during the pregnancy. Severity of each stressor was also recorded using an established ranked scale of typical stressor intensities (21, 57).

**Table 1:** Comparison groups.

**Prenatal stress**	**SERT genotype**	***N***	**Group**
High	SS	6	G1a
	LS	11	G1b
	LL	5	G2a
Low	SS	6	G2b
	LL	6	G3

*Samples were divided into five groups based on SERT genotypes and prenatal stress level*.

### miRNA Expression Profiling

Lymphocytes from all mothers, varying in *SERT* genotype and degree of stress exposure, were assessed for miRNA expression. Total RNAs were isolated using the mirVana kit (Ambion, Foster City, CA, USA) to capture small RNAs. miRNA profiling was conducted using a previously used service provider (LC Sciences, Houston, TX, USA), which applies an in-house-developed μParaflo® technology platform. Each region of chips used consists of miRNA probes, which detect miRNA transcripts listed in Sanger miRBase Release 21 (http://www.mirbase.org/). Multiple control probes were included on each chip for quality controls of chip production, sample labeling, and assay conditions. Image digitization was done using “Array-Pro Analyzer” (Media Cybernetics, Rockville, MD, USA). Normalization was carried out using LOWESS (locally weighted scatterplot smoothing), a pair-wise regression method, on background-subtracted data. The purpose of the experiment was to identify miRNAs that differ in expression levels (DE miRNAs) among the comparison groups. ANOVA was applied to detect DE miRNAs (*p* < 0.05), considering the variability of the expression levels across all samples (within and among the groups). miRDB was used to identify miRNA predicted target genes (82). Pathway analysis was conducted using the DAVID tool (83).

### Exome Sequencing

To explore other effects of maternal genotype, the same service provider was used to run whole-exome sequencing (WES). The Illumina HiSeq 2500/4000 platform was used with 50 × coverage depth. Data were analyzed using GATK (Genome Analysis Toolkit) and applying an analytical pipeline that included removing low-complexity sequences, aligning of sequence against reference human genome, excluding duplicate reads, sorting nucleotide sequence alignment, and base quality score and variant calling [INDEL and single-nucleotide variants (SNVs)]. Annotation and filtering of variants included minor allele frequency filtration (MAF < 0.05 using databases such as dbSNP, 1000 Genome, TopMed, and ExAC). False-positive variants commonly seen in WES were sorted out and removed, using recommendation by Fuentes Fajardo et al. (84). The functional effect of variants was assessed using prediction programs (SIFT and PolyPhen2), for retained variants with MAF < 5% and residing within the coding regions or 10 bases upstream/downstream from splicing junctions. Variants were divided into three categories based on their predicted effect on the protein function, with the following description: high-impact effects (splice site acceptor, splice site donor, start lost, exon deleted, frame shift, stop gained, stop lost), moderate-impact effects (non-synonymous coding, codon change/insertion/deletion, UTR 5′/UTR 3′ deletion), and low-impact effects (synonymous/non-synonymous start/stop, start gained, synonymous coding). We focused on high-impact variants.

## Results

MicroRNA expression in blood samples (*n* = 34) from mothers of children with ASD, with known pregnancy stress history, was profiled. Comparisons were conducted based on *SERT* genotypes (LL, LS, and SS) and prenatal stress level (high vs. low). Among the 2,500 mature miRNAs examined in all five groups, 119 miRNAs were found to be differentially expressed (DE). Ninety of the DE miRNAs (76%) showed a different pattern of expression in high vs. low stress-exposed groups (suggestive of being stress-dependent miRNAs); 77 (86%) of them were upregulated by stress and 13 (14%) were downregulated in the high- vs. low-stress groups, as shown in Supplementary Table 1A. Out of these DE miRNAs, the following three have been previously reported in association with stress: miR-1224-5p and miR-331-3p were found to be stress-dependent in offspring mouse brains by our group (78), and miR-145-5p has been reported in association with maternal stress (71, 85). Our previous work had suggested that prenatal stress exposure interacts with maternal stress susceptibility associated with the *SERT* genotype. To further explore this interaction, miRNA profiles were also assessed in the three groups exposed to a high level of prenatal stress, stratified by *SERT* genotypes. This analysis detected a smaller number of DE miRNAs (*n* = 20), as shown in Supplementary Table 1B. Moreover, five out of 20 (miR-663a, miR-664b-5p, miR-4787-5p, miR-6125, and miR-7704) were shared with the stress-dependent miRNAs, making them potential candidates for the *SERT*/stress mechanism.

For target predictions, we prioritized those DE miRNAs that are most likely to be associated with this G × E interaction model. To do so, we compiled a list of eight DE miRNAs (Table 2), including the five candidates for stress × *SERT* genotype interactions and the three that have been previously associated with stress, discussed above. A total of 1,074 genes were predicted for these eight DE miRNAs. Two hundred thirteen of them were annotated in the OMIM database. The leading functional target for these genes was the dopaminergic synapse, congruent with our effects on dopamine in the striatum described below in the gene × stress model (86), in addition to pathways associated with addiction and other neurotransmitter systems including glutamate, GABA, the serotonergic system, and the cholinergic system (Table 3). Additionally, *DYRK1A* was a predicted target for three of these miRNAs (miR-1224-5p, miR-145-5p, and miR-663a).

**Table 2:** Eight DE microRNAs candidate for stress × SERT interactions (stress upregulated = red, downregulated = blue^c^).

		**SERT genotypes**				
		**High stress**			**Low stress**	
**microRNA**	***P*-value**	**SS**	**LS**	**LL**	**SS**	**LL**
hsa-miR-6125	0.000009	3,952	7,249	5,973	1,854	2,854
hsa-miR-4787-5p	0.00002	3,086	5,952	6,561	1,601	2,325
hsa-miR-663a	0.00002	100	242	177	38	70
hsa-miR-7704	0.0001	6,175	11,183	7,739	2,281	4,289
hsa-miR-1224-5p^a^	0.001	1,491	1,814	1,663	309	485
hsa-miR-664b-5p	0.002	308	548	787	115	80
hsa-miR-331-3p^a^	0.002	363	457	398	976	1,018
hsa-miR-145-5p^b^	0.01	209	387	471	675	660

*See Table 1 for grouping and sample sizes^c^*.

a *Exhibited a stress-dependent expression pattern in rodent brain samples from embryos exposed to prenatal stress (78)*.

b *Has been reported in association with maternal stress (71, 85)*.

c *Numbers represent normalized average expression level (signal intensity on the array)*.

**Table 3:** Functional annotation of the predicted target genes for eight DE microRNAs listed in Table 2.

**Term**	**Count**	***P*-value**	**Fold enrichment**	**Bonferroni**	**Benjamini**	**FDR**
Dopaminergic synapse	22	5.46E-06	3.1	0.001	0.001	0.007
Amphetamine addiction	14	3.82E-05	3.9	0.010	0.003	0.050
Cocaine addiction	11	3.32E-04	4.0	0.081	0.012	0.432
Glutamatergic synapse	16	1.22E-03	2.6	0.267	0.026	1.582
Circadian entrainment	14	2.04E-03	2.7	0.404	0.036	2.619
Cholinergic synapse	15	3.40E-03	2.4	0.579	0.053	4.337
Alcoholism	16	6.76E-03	2.2	0.821	0.069	8.455
Serotonergic synapse	14	7.52E-03	2.3	0.853	0.071	9.368
GABAergic synapse	12	7.94E-03	2.5	0.868	0.072	9.861

*Using miRDB a total of 1,074 target genes were predicted for the DE microRNAs and DAVID was used for functional annotation of these genes. Count refers to the number of genes from the input list (i.e., predicted targets for the eight DE miRNAs) annotated with a given term*.

Furthermore, we found that high-stress groups exhibited a notably elevated level of high-impact SNVs compared with the low-stress groups, as shown in Supplementary Table 2. When comparing groups in the two extreme ends (G1a vs. G3), total number of SNVs per subjects were 4.9 times more in high-stress-exposure mothers with the SS genotype than those with low-stress-exposure and the LL genotype. SNVs seen in more than one subject in each group are listed in Table 4. In this list, we prioritized SNVs identified in G1a, the high-risk genotype (SS) group. Recurrent variants in four genes, *AMY1C, CPA4, GC*, and *NOTCH2NL*, were observed in this group. The *NOTCH2NL* and *AMY1C* variants were also detected in the LS and LL genotype groups, respectively.

**Table 4:** High-impact recurrent SNVs identified in each group.

**Prenatal stress level**	**Gene**	**Variant**	**Chromosome**	**Group**	**Subject ID**	**Annotation**	**HGVS.c**	**HGVS.p**	**SERT genotype**
High	AMY1C	rs140363602	chr1	G1a	10M, 51M	stop_gained	c.1054C>T	p.Arg352*	SS
	CPA4	rs145012020	chr7	G1a	10M, 25M	stop_gained	c.777G>A	p.Trp259*	
	GC	rs76781122	chr4	G1a	10M, 51M	start_lost	c.3G>T	p.Met1?	
	NOTCH2NL	rs140871032	chr1	G1a	10M, 44M	stop_gained	c.220C>T	p.Arg74*	
	NOTCH2NL	rs374113588	chr1	G1a	10M	stop_gained	c.154C>T	p.Arg52*	
	KIAA1919	rs117505745	chr6	G1b	32M, 53M	stop_gained	c.545T>A	p.Leu182*	LS
	LILRA1	rs150508449	chr19	G1b	23M, 31M	stop_gained	c.781G>T	p.Gly261*	
	LRRC9	rs368587449	chr14	G1b	32M, 48M	stop_gained	c.3781C>T	p.Arg1261*	
	LRRC9	rs35427175	chr14	G1b	45M	stop_gained	c.3113G>A	p.Trp1038*	
	NOMO2	rs200294351	chr16	G1b	28M, 31M, 53M	stop_gained	c.2122G>T	p.Glu708*	
	NOTCH2NL	rs140871032	chr1	G1b	22M, 46M	stop_gained	c.220C>T	p.Arg74*	
	OLFM4	rs34067666	chr13	G1b	12M, 22M	stop_gained	c.640C>T	p.Arg214*	
	RHBDD3	rs138870856	chr22	G1b	23M, 48M	stop_gained	c.867G>A	p.Trp289*	
	SULT1C3	rs112050262	chr2	G1b	46M, 53M	stop_gained	c.108G>A	p.Trp36*	
	VCX3B	rs5978242	chrX	G1b	23M, 46M	splice_donor_variant	c.387+1G>C	NA	
	AMY1C	rs140363602	chr1	G2a	27M, 43M	stop_gained	c.1054C>T	p.Arg352*	LL
	PRSS1	rs147366981	chr7	G2a	13M, 27M	stop_gained	c.166C>T	p.Gln56*	
	SULT1C3	rs112050262	chr2	G2a	27M, 43M	stop_gained	c.108G>A	p.Trp36*	
Low	NIT1	rs76502631	chr1	G2b	04M, 21M	splice_donor_variant	n.96+1G>A	NA	SS
	CBWD1	rs199631831	chr9	G3	29M	splice_acceptor_variant	c.2961C>G	p.Tyr987*	LL
	CBWD1	rs199901774	chr9	G3	15M	splice_donor_variant	c.816+1G>T	NA	

*Functional effect of variants was assessed using SIFT and PolyPhen2 programs*.

## Discussion

Our previous work supported a specific gene and stress interaction in the development of ASD (57). The present study provides evidence for epigenetic alterations in relation to a promising G × E model (prenatal maternal stress × *SERT* gene) in ASD detected in maternal blood samples. These findings are remarkable as these changes were detected in samples from mothers several years after stress-exposed pregnancies. Previous work has demonstrated an altered miRNA expression in the brains of offspring mice exposed to maternal stress and altered maternal serotonin transporter genotype (heterozygous KO) (78), with some of the same miRNA differentially expressed in the present study with human maternal blood. Additionally, DAVID revealed that the leading functional target of the miRNA differentially expressed by G × E in the maternal clinical samples was the dopaminergic synapse. This is of particular interest with the growing interest in the role of dopamine in social behavior (87), and the potential role for this as a treatment target in this subset of cases with ASD. The effects on dopaminergic synapse targets are also of particular interest given our recent finding that prenatal stress exposure in *SERT*-het mice resulted in increased striatal DA in offspring brains (86). Furthermore, these dopaminergic changes were reversed with DHA (86). The effects on glutamatergic and GABAergic targets are of particular interest in autism, given the importance of the excitatory/inhibitory imbalance in ASD (88), and the effects on serotonergic targets would be anticipated given the inclusion of maternal *SERT* genotype in the G × E interaction.

Persistent miRNA changes have been observed previously in other conditions, such as after cessation of smoking (89). Thus, the maternal miRNA changes observed after prenatal stress exposure associated with ASD appear to be long-lasting. This is an important step forward in our understanding of ASD, whose incidence appears to continue to rise, by identifying potential markers for an etiological subtype. Future work should explore these markers in children as well. Regulation of miRNA stability is currently an underappreciated area of research, but better understanding of its mechanism is likely to contribute to a broader range of regulation that can impact gene expression. New areas of investigation into how gene expression can be controlled by miRNA stability may provide novel advancements in therapeutic applications related to the fluctuations of gene expression associated with human disorders (90).

The finding that *DYRK1A* was targeted by three different DE miRNAs is of interest. *DYRK1A* was identified as a strong risk candidate gene for ASD based on a combination of recurrent *de novo* likely gene-disruptive mutations in affected individuals and their absence/very low frequency in controls (91). *De novo* dominant mutations in *DYRK1A* substantially reduce kinase function and account for ≈0.5% of severe developmental disorders (92). *DYRK1A* was found to be downregulated in samples from women exposed to Superstorm Sandy during pregnancy, across all trimesters (93). Overexpression of *Dyrk1a* causes a major deficit in the level of serotonin in the brain, as well as deficit of dopamine and adrenaline neurotransmitters in a transgenic mouse model for Down syndrome (94).

The exploratory exome sequencing revealed some findings of potential interest. First, the finding of *NOTCH2NL* variants in four individuals with high stress exposure is of particular interest due to its critical role in cortical development and radial glial stem cell proliferation, as well its association with the 1q21.1 distal deletion/duplication syndrome, where duplications are associated with autism (95). Additionally, the finding of *AMY1C* variants in four individuals with high stress exposure is of interest since salivary amylase is significantly correlated with repetitive behaviors in individuals with developmental disorders (96). Among the other SNVs, one variant, rs76781122 in *GC*, detected in two subjects from the high-stress-exposure group, in particular, caught our attention. This variant alters the start codon and creates a stop codon. The *GC* gene encodes a vitamin D-binding protein (VDBP), the major plasma carrier of vitamin D metabolites. A non-synonymous variant, R21L, in this gene has been reported in association with migraine, a condition often triggered by stress factors (97). Several SNPs in *GC* have been shown to effect vitamin D levels (98). The VDBP has also been associated with inflammatory-mediated conditions (99). Notably, vitamin D receptors are highly abundant in brain and involved in several key biological processes, including the serotonin-mediated pathways (100). One possible implication is that pregnant mothers, carriers of rs76781122, may have had decreased levels of VDBP, which further places them at risk for a compromised immune system in a stressful environment. When comparing the results from miRNAs profiling and WES, it was noted that six out of 1,047 predicted target genes (*MADD, EPS15, PCDHGA10, WWOX, PGPEP1L*, and *NDUFA10*) for the DE miRNAs (miR-1224-5p, miR-145-5p, miR-7704, miR-663a, miR-664b-5p, and miR-6125, respectively), also harbor SNVs in the high-stress groups. Further investigations are warranted to assess potential functional relation between these genes and the miRNAs.

Stress precipitates a systemic physiological response that involves inflammatory, cellular, and metabolic processes and their epigenetic regulation. Epidemiological studies have found increased susceptibility to schizophrenia, ASD, or ADHD to be associated with prenatal stress exposure. While exact pathophysiological mechanisms are still unknown, maternal immune activation, alteration of the hypothalamic–pituitary–adrenal axis, and epigenetic modifications regulating gene expression were proposed as potential causes of neuronal proliferation and migration disturbances in the developing fetus, which may lead to the increased susceptibility to these disorders (101, 102). Multiple studies using human whole blood reported that stress may cause shifts in the concentration levels of specific miRNAs and suggest their potential use as biomarkers in human whole blood (103–105).

While maternal exposure to stress during pregnancy is an elusive risk factor contributing to a wide range of ASD-like traits in offspring, a recent study involving children with ASD revealed that exposure to gestational stress can be used as a strong predictor of severity of ASD symptoms (*p* = 0.048) and communication abilities (*p* = 0.004), even after controlling for other variables. Moreover, significant increases in symptom severity were seen with multiple (two or more) prenatal stressful life events (29).

Recent research evidence suggests that miRNAs are both responsive and susceptible to significant environmental insults such as gestational stress and may increase the offspring vulnerability to stress-related psychopathological conditions (85).

The exact route through which maternal stress affects gene expression in the offspring's brain are not yet known, but involvement of epigenetic changes may be one such mechanism (106). Epigenetic processes such as microRNAs are among gene regulatory mechanisms that are influenced by environmental factors such as stress, and identifying their potential mis-regulation will contribute to narrowing the existing gap in our understanding of the mechanism of maternal stress and ASD.

The findings from the exome sequencing, while exploratory in nature, deserve further investigation. It will be critical to determine whether aspects of vitamin D metabolism, known to have a range of effects on offspring during development (107), might also contribute to developmental susceptibility to the effects of prenatal stress, or if the other SNVs might have critical salience, such as the potential for altered stress reactivity among mothers with *AMY1C* variants (96).

To our knowledge, this study is the first to examine a potential mechanism for this specific interaction between genetics and stressors during a specific prenatal period. These findings may serve as evidence of a biomarker for this mechanism, or possibly a common biomarker for several etiologies, which warrants further investigation. As numerous genes as well as *SERT* can affect stress reactivity, exploring epigenetic pathways by which this occurs more broadly will help to identify pathways of action regardless of the specific gene associated with stress exposure. Additionally, it will be critical for subsequent studies to examine the impact of other *SERT* variants, including the L(A) allele (108, 109), as well as others (110, 111). It will also be critical to better understand the time course of development of these markers, from at the time of initial stress exposure through birth, to determine other such markers that might be present earlier in the course. While the apparent persistence of these stress-associated miRNA changes is remarkable, and there is precedent for persistent miRNA changes in other settings (89), one cannot be certain as to the relationship here, and future studies will need to reevaluate these findings at an earlier time point, and eventually during pregnancy in a longitudinal study. However, this would not appear to be due to changes simply resulting from raising a child with ASD, since all samples were collected from mothers of children with ASD, and the miRNA findings herein are due to the effects of the G × E interaction within the population of mothers of children with ASD. We cannot exclude, though, the possibility that other maternal factors, may have contributed such as those identified in the exome sequencing, or it might be due to stress resulting from phenotypical differences in the G × E-associated cases, since previous work suggests that prenatal stress-associated autism can be more severe (29). Thus, future work will be needed to determine whether these miRNA findings might serve as a biomarker that could be targeted in approaches to mitigate the effects of prenatal stress.

## Data Availability Statement

The datasets presented in this study can be found in online repositories. The names of the repository/repositories and accession number(s) can be found below: NCBI Gene Expression Omnibus, accession no: GSE179222.

## Ethics Statement

The studies involving human participants were reviewed and approved by University of Missouri Health Sciences Institutional Review Board. The patients/participants provided their written informed consent to participate in this study.

## Author Contributions

DQB and ZT conceptualized and designed the study, coordinated and supervised data management, drafted the initial manuscript, and reviewed and revised the manuscript. ZT led the epigenetics analysis. DQB led the sample and clinical aspects. AS implemented the epigenetics analysis with ZT. AJ assisted with WES data analysis and literature review. DB assisted MT and PH with the genotyping. JN-M assisted with statistical data analysis and interpretation. PH obtained the clinical samples, stress surveys, and with DQB. MT oversaw the genetics for the samples. PH and MT also reviewed and revised the manuscript. BF coordinated all aspects of the between-site collaboration and reviewed and revised the manuscript. All authors approved of the final manuscript as submitted.

## Conflict of Interest

DB has been a consultant or on the advisory board for Yamo Pharmaceuticals, Stalicla Biosciences, Impel Pharmaceuticals, MA Pharmaceuticals, and Quadrant Biosciences, which are all unrelated to this work. The remaining authors declare that the research was conducted in the absence of any commercial or financial relationships that could be construed as a potential conflict of interest.

## Abbreviations

ANOVA: analysis of variance
ADHD: attention deficit hyperactivity disorder
ADI-R: Autism Diagnostic Interview-Revised
ADOS: Autism Diagnostic Observation Scale
ASD: autism spectrum disorder
DNA: deoxyribonucleic acid
DOHaD: developmental origins of health and disease
DE: differentially expressed
G x E: Gene x Environment
GATK: Genome Analysis Toolkit
IGFBP-1: insulin-like growth factor-binding protein 1
LOWESS: locally weighted scatterplot smoothing
L: long
miRNA and micro RNA: micro ribonucleic acid
MAF: minor allele frequency
PPARα: peroxisome proliferator-activated receptors α
OGT: O-GlcNAc transferase
PCR: polymerase chain reaction
*SERT*: serotonin transporter
S: short
SNP: single nucleotide polymorphisms
SNVs: Single Nucleotide Variants
WES: Whole Exome Sequencing
wt: wild type.
