# Supplementary material for: microRNAs and Gene–Environment Interactions in Autism: Effects of Prenatal Maternal Stress and the SERT Gene on Maternal microRNA Expression
Source: Front Psychiatry. 2021 Jul 5;12:668577. doi: 10.3389/fpsyt.2021.668577 (PMC8288023; doi:10.3389/fpsyt.2021.668577)
Supplement: Supplementary file 2 [file Table_2.DOCX]

**Note:** When comparing groups in the two extreme ends (G1a vs G3), total number of SNVs per group/subjects (8.8 vs 1.8) were 4.9 times more in high stress exposure mothers with the SS genotype than those with low stress exposure and the LL genotype.

**Variant Calling and Analysis**

-SNP and INDEL sites (SNVs) were analyzed using GATK.

**Step 1 Genome Annotation**

**SNP Results**

-SNP location classification and annotation

- Class (synonymous coding, non-synonymous coding, stop gained, stop lost, start gained, start lost)
- Position (downstream, exon, intergenic, intron, splicing, upstream, UTR 3’ prime, UTR 5’ prime)

-Effect of mutations on the genome

- High-Impact Effects (splice site acceptor, splice site donor, start lost, exon deleted, frame shift, stop gained, stop lost)
- Moderate-Impact Effects (non-synonymous coding, codon change/insertion/deletion, UTR 5’/UTR 3’ deletion)
- Low-Impact Effects (synonymous start, non-synonymous start, start gained, synonymous coding, synonymous stop, non-synonymous stop)

**INDEL Results**

-INDEL location classification and annotation

- Class (codon change plus codon deletion, codon change plus codon insertion, codon deletion, codon insertion, frame shift, frame shift plus stop gained)
- Position (downstream, exon, intergenic, intron, splicing, upstream, UTR 3’ prime, UTR 5’ prime)

**Step 2. Coding Region Annotation**

- Variants within the coding region or within the upstream/downstream 10 bases region from the splicing junction are retained as candidate sites that may cause diseases

**Step 3. Protein Function Annotation**

Functional effect of variants was assessed using prediction programs (SIFT and PolyPhen2), for retained variants.

1. The effect of amino acid substitutions on protein function was predicted [SIFT tool]
2. It determines whether the amino acid substitutions are functionally neutral or deleterious (score > 0.05 = the mutation is tolerable, score < 0.05 = the mutation is harmful, and has greater impact on protein function).
3. PolyPhen2 (Polymorphism Phenotyping) is a tool for predicting the effect of amino acid substitutions on protein structure and function (PolyPhen2 values > 0.95 = the mutation site has a great impact on gene function)
